# Ultra-Fine Control of Silica Shell Thickness on Silver Nanoparticle-Assembled Structures

**DOI:** 10.3390/ijms222111983

**Published:** 2021-11-05

**Authors:** Eunil Hahm, Ahla Jo, Eun Ji Kang, Sungje Bock, Xuan-Hung Pham, Hyejin Chang, Bong-Hyun Jun

**Affiliations:** 1 Department of Bioscience and Biotechnology, Konkuk University, Seoul 05029, Korea; greenice@konkuk.ac.kr (E.H.); iamara0421@konkuk.ac.kr (A.J.); ejkang@konkuk.ac.kr (E.J.K.); bsj4126@konkuk.ac.kr (S.B.); phamricky@konkuk.ac.kr (X.-H.P.); 2 Division of Science Education, Kangwon National University, Chuncheon 24341, Korea

**Keywords:** silica shell, fine control, shell thickness, assembled structures

## Abstract

To study the distance-dependent electromagnetic field effects related to the enhancement and quenching mechanism of surface-enhanced Raman scattering (SERS) or fluorescence, it is essential to precisely control the distance from the surface of the metal nanoparticle (NP) to the target molecule by using a dielectric layer (e.g., SiO_2_, TiO_2_, and Al_2_O_3_). However, precisely controlling the thickness of this dielectric layer is challenging. Herein, we present a facile approach to control the thickness of the silica shell on silver nanoparticle-assembled silica nanocomposites, SiO_2_@Ag NPs, by controlling the number of reacting SiO_2_@Ag NPs and the silica precursor. Uniform silica shells with thicknesses in the range 5–40 nm were successfully fabricated. The proposed method for creating a homogeneous, precise, and fine silica coating on nanocomposites can potentially contribute to a comprehensive understanding of the distance-dependent electromagnetic field effects and optical properties of metal NPs.

## 1. Introduction

Core-shell nanomaterials comprising an inner core and an outer shell have attracted considerable attention due to their properties derived from both the core and the shell materials [1,2,3,4,5]. It has been reported that the surface reactivity [6,7,8,9], thermal stability [10,11], colloidal stability [12,13], dispersibility [14,15], absorption [16,17,18], chemical configuration transition [19,20], and releasing properties [21,22] of core-shell nanomaterials mostly depend on the chemical and physical properties of the shell material. Various encapsulation methods have been extensively studied to prepare core-shell hybrid nanomaterials with excellent functionalities [2].

Shell materials, such as metals [23,24,25], silica [26,27,28], polymers [29,30], and glucose [31,32], are often chosen to extend the applications of nanoparticles (NPs). Among these shell materials, silica has been used as an ideal material for preparing core-shell nanomaterials, for example, silica based-metals, -ceramics, -semiconductors, and -magnetics [33], due to its low cost and its excellent properties, such as easy surface modification, optical transparency, chemical inertness, biocompatibility, and easy bio-binding with functional groups [33,34].

The thickness of the silica coating on NPs considerably impacts the physico-chemical properties of core-shell NPs. Phenomena such as metal-enhanced fluorescence (MEF) and surface-enhanced Raman scattering (SERS), which occur on the surfaces of metal NPs due to the intense plasmon-induced electric field, depend strongly on the distance between the metallic surfaces and the coating molecules. Therefore, accurate and fine control of the silica coating of the nanocore particles is necessary. The shell thickness of magnetic NPs affects the distance dependent magnetic field properties of the NPs. However, previous studies on the control of silica shell thickness were mainly conducted on single metal NPs, and there are few studies on the extensive fine control and uniformity of the shell [35,36,37,38]. In addition, complicated composite NPs as a core-multi-shell NPs with multiplex functions have been studied recently. The disorder of chemical configuration during synthesis leads to a variety of core-multi-shell nanomaterials [20]. Therefore, it is also necessary to study a method for the fine-thickness-control of the coating of composite NPs rather than single metal NPs.

Our research group has developed several silver NP-assembled silica (SiO_2_@Ag) NPs that exhibit multiple properties, such as fluorescence, magnetism, and SERS [39,40]. The as-prepared NPs showed strong and reproducible Raman enhancement and/or fluorescence at a single NP level [41,42,43,44]. In this study, we present a new approach to obtain a fine silica shell coating on composite SiO_2_@Ag NPs (SiO_2_@Ag@SiO_2_). The silica shell thickness was finely controlled in the range 5–40 nm by adjusting two parameters: the number of NPs and the amount of silica precursor (sodium silicate (Na_2_SiO_3_) and tetraethylorthosilicate (TEOS)) during the silica coating process. This simple method resulted in the formation of a uniform silica shell on the surface of the SiO_2_@Ag NPs. This technique is expected to be useful in the optimization of the fluorescence and the SERS signal of SiO_2_@Ag NPs and opens up an opportunity for the fine control of the silica shell thickness of other nanocomposites.

## 2. Results and Discussion

**Figure 1:**
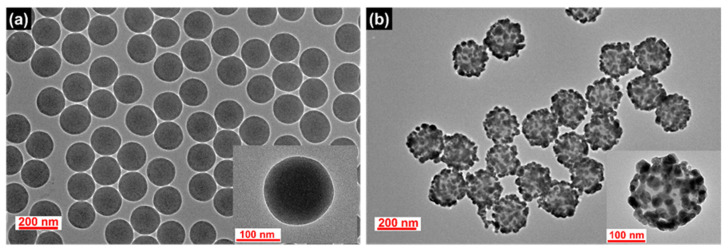
TEM images of the synthesized (**a**) SiO_2_ NPs and (**b**) SiO_2_@Ag NPs.

SiO_2_@Ag NPs were prepared as a nanocomposite prior to the silica shell coating. SiO_2_ NPs, a template for the deposition of Ag NPs, were synthesized by the Stöber method [45]. The surface of the SiO_2_ NPs was modified with thiol groups by incubating the NPs with 3-mercaptopropyl trimethoxysilane (MPTS), and then Ag NPs were introduced on the SiO_2_ surface by the in situ reduction of Ag^+^ ions with a reducing agent (octylamine). The TEM images of the SiO_2_ NPs and the resultant SiO_2_@Ag NPs are shown in Figure 1. The SiO_2_ and SiO_2_@Ag NPs were found to be homogeneous and well dispersed without aggregation. The average diameter of the SiO_2_ NPs is 153 ± 2.4 nm as shown in Figure 1a. After the assembling of the Ag NPs, the average diameter of SiO_2_@Ag NPs increased up to 192 ± 7.5 nm, and the Ag NPs with an average size of 21.5 ± 6.1 nm were densely assembled on the SiO_2_ surface (Figure 1b). As shown in Figure S1, the absorption intensity and the extinction maxima of the plasmonic resonance bands significantly changed after the assembly of the Ag NPs on the SiO_2_ NPs surface. Broad absorption in the range from 322 to 800 nm with a maximum peak at 430 nm was exhibited by the SiO_2_@Ag NPs suspension, indicating that Ag NPs aggregated on the surface of SiO_2_ NPs [24], which is consistent with the results shown in Figure 1.

**Figure 2:**
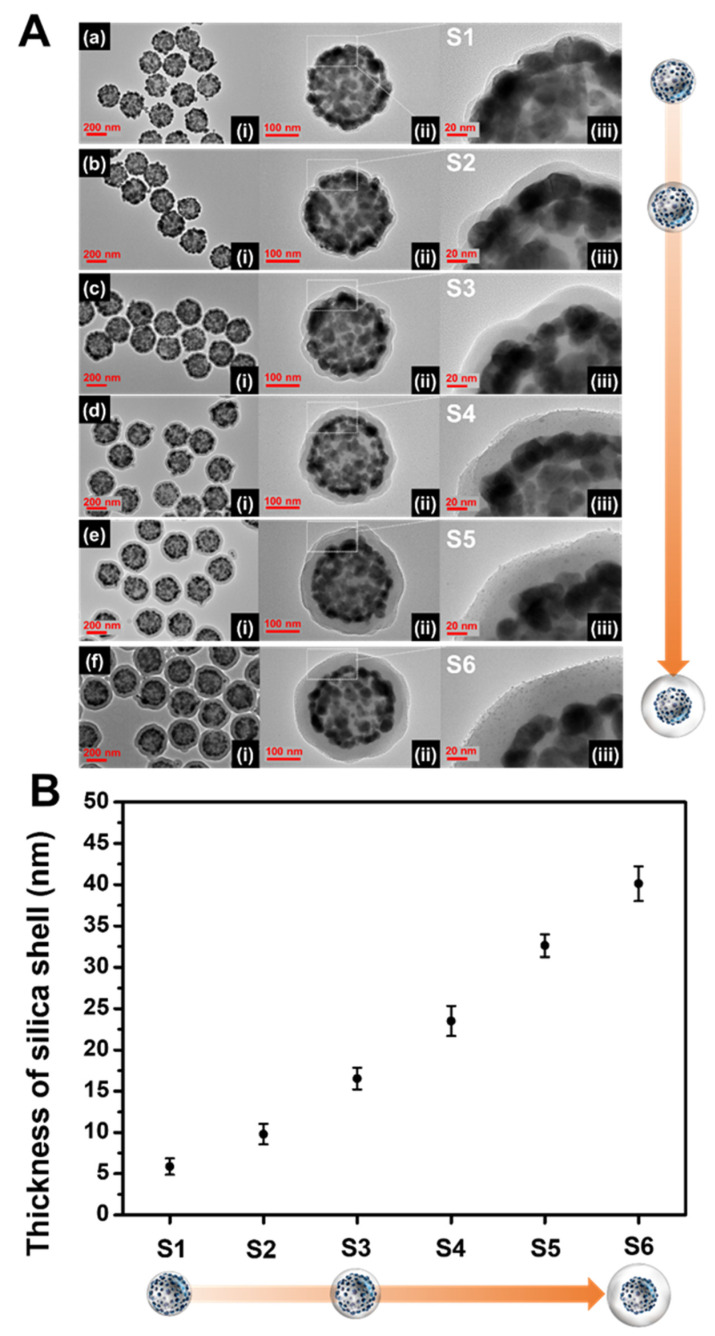
(**A**) TEM images at (i) low and (ii, iii) high magnifications. (**B**) The thickness of the silica shell layer on the surface of the SiO_2_@Ag NPs synthesized under various conditions (**a**–**f**).

The thickness of the SiO_2_ shell is highly sensitive to the experimental conditions, which may generate inaccurate and unstable results. It is difficult to evenly form a silica shell with sub-nanometer thickness on the NPs because excessive shell-forming reactions should be excluded to prevent the formation of non-core silica structures such as silica NPs. In our procedure, Na_2_SiO_3_ as a silica precursor was added to the aqueous SiO_2_@Ag NPs solution, and then the aqueous dispersion was transferred into ethanol. The solvent exchange procedure serves to precipitate the silicate portion (monomer or oligomer) remaining in the solution due to a sharp decrease in solubility, forming a silica shell [46,47]. One would expect that a thicker silica shell would form as the amount of Na_2_SiO_3_ is increased. However, experimental results showed that the thick silica shell formation was difficult, and new non-core SiO_2_ NPs were generated (Figure S2). On the other hand, it was found that the number of SiO_2_@Ag NPs is a key parameter in controlling the thickness of the silica shell (Figure 2). The amount of SiO_2_@Ag NPs added was adjusted by changing the quantity of SiO_2_ (5, 10, and 20 mg), while the amount of Na_2_SiO_3_ was fixed at 90 µmol. The average number of SiO_2_ NPs per mg was calculated to be 2.01 × 10^11^ ± 9.35 × 10^9^. Therefore, the average number of 5, 10, and 20 mg SiO_2_ are 1.01 × 10^12^; 2.01 × 10^12^, and 4.02 × 10^12^, respectively (Table S1). The resultant silica shell thickness of each sample was 5.8 ± 0.9 nm (S1), 9.7 ±1.2 nm (S2), and 16.5 nm ±1.3 (S3), which correspond to 4.02 × 10^12^ SiO_2_ NPs, 2.01 × 10^12^ SiO_2_ NPs, and 1.01 × 10^12^ SiO_2_ NPs, respectively (Figure 2a–c). When the number of SiO_2_@Ag NPs is doubled (from 1.01 × 10^12^ NPs (S3) to 2.01 × 10^12^ NPs (S2), and from 2.01 × 10^12^ NPs (S2) to 4.02 × 10^12^ NPs (S1)), the thickness of the silica shell decreased by 6.8 and 3.9 nm, respectively. Therefore, the thickness of the silica layer in S1 to S3 was observed to increase as the number of nanoparticles decreased. The shell thickness is inversely proportional to the cubic root of the number of nanoparticles as shown in the following equation:

(1) $${\mathrm{SiO}}_{2}\;\mathrm{shell}\;\mathrm{thickness}=\frac{\left[2\times \sqrt[{3}]{\left\{\left(\frac{n\times M}{\;\rho \times N}+V\right)\times \frac{3}{4\pi }\right\}}-d\right]}{2}$$

where n is the number of mols of Na_2_SiO_3_ or TEOS, M is the molecular weight of SiO_2_, ρ is the density of SiO_2_, N is number of NPs, V is the volume of SiO_2_@Ag, and d is the diameter of SiO_2_@Ag. Indeed, the thicknesses of the silica shells of the nanoparticles (S1 to S3) were found to be consistent with the calculated silica shell values in Table S2, even though the observed values were thicker than the calculated values (~1.5–1.6 nm). 

Although the thickness control of the silica shell was successful as described above, reducing the number of SiO_2_@Ag NPs leads to a low yield of the SiO_2_@Ag@SiO_2_ product. To generate a thick silica shell coating on the surface of SiO_2_@Ag NPs with a desired and reasonable product yield, a secondary silica shell precursor was added into the suspension of SiO_2_@Ag NPs coated with silica and Na_2_SiO_3_. Tetraethyl orthosilicate (TEOS) was used as a secondary silica shell precursor in the presence of NH_4_OH (as base catalyst in hydrolysis of TEOS). Homogeneous silica shells with variable thickness were grown on SiO_2_@Ag NPs by the addition of different volumes of TEOS. TEOS was added into the S2 suspension (SiO_2_@Ag NPs coated by Na_2_SiO_3_). TEOS volumes of 88.7, 177, and 266 μmol were added into the suspension, which corresponded to the final TEOS concentration of 1.16, 2.33, and 3.49 mM, respectively. Figure 2d–f shows the TEM images of the SiO_2_@Ag@SiO_2_ NPs generated by controlling the molar concentration of TEOS in the suspension. The silica shell thicknesses of the SiO_2_@Ag@SiO_2_ NPs were obtained to be 23.5 ±1.8 nm (S4), 32.6 ±1.3 nm (S5), and 40.1 ± 2.1 nm (S6). The thickness of the silica shell increased by approximately 7–9 nm as the final concentration of TEOS increased from 1.16 to 3.49 mM. The observed silica thicknesses of S4–S6 were thicker than those of the calculated values (Table S2). In particular, the ratio of the observed and calculated values of the silica shell thickness was consistent and was higher when TEOS was added to the suspension. Based on these results, we concluded that the number of SiO_2_@Ag NPs is effective for controlling thin silica coatings and the addition of TEOS is effective for controlling thick silica coatings of SiO_2_@Ag NPs. The absorbance spectra of SiO_2_@Ag@SiO_2_ NPs are shown in Figure S3. The spectra of thin silica coated SiO_2_@Ag NPs were insignificantly different from that of the SiO_2_@Ag NPs, indicating that no leakage of Ag NPs occurred from the surface of the SiO_2_@Ag NPs during the silica shell coating. As expected, a thin silica shell coating does not seriously affect the plasmonic resonance properties of thin shell silica coated SiO_2_@Ag NPs [38]. To demonstrate the stability during storage, SiO_2_@Ag@SiO_2_ NPs were stored in EtOH and water for 10 days. The shapes of both SiO_2_@Ag@SiO_2_ NPs synthesized using Na_2_SiO_3_ with or without TEOS did not show any significant differences after 10 days in water and EtOH (Figure S4). This study indicates that the SiO_2_@Ag@SiO_2_ NPs using Na_2_SiO_3_ with or without TEOS are stable when stored in water and EtOH.

## 3. Materials and Methods

### 3.1. Materials

Tetraethylorthosilicate (TEOS), ethyl alcohol (EtOH, 99.5% and 95%), 3-mercaptopropyl trimethoxysilane (MPTS), silver nitrate (AgNO_3_), polyvinylpyrrolidone (PVP, MW 40,000), ethylene glycol (EG), octylamine (OA), hydrochloric acid (HCl), sodium hydroxide (NaOH), and acetone were purchased from Sigma-Aldrich (St. Louis, MO, USA) and used without further purification. Aqueous ammonium hydroxide (NH_4_OH, 27%) was purchased from Daejung (Siheung, Gyeonggi-do, Korea). Water was purified using a Direct-Q Millipore water purification system (SAM WOO S&T Co., Ltd., Seoul, Korea).

### 3.2. Methods

#### 3.2.1. Synthesis of Ag-Embedded Silica Nanoparticles (SiO_2_@Ag NPs)

SiO_2_ NPs (~153 nm) were synthesized using the modified Stöber method. TEOS (1.6 mL) was added to EtOH (40 mL) in a round flask. Then, NH_4_OH (3 mL) was added quickly to this solution. The mixture was vigorously stirred at 700 rpm for 20 h at room temperature (RT). Then, the mixture was centrifuged (8500 rpm, 15 min) and washed 3 times with EtOH to remove the excess reagents. After washing, the SiO_2_ NPs were dispersed in EtOH, and the SiO_2_ concentration was adjusted to 50 mg·mL^−1^.

To embed Ag NPs on the SiO_2_ surface, the SiO_2_ NP suspension was incubated with MPTS to transfer the hydroxyl groups on its surface to the thiol groups. In particular, the SiO_2_ NPs (50 mg·mL^−1^, 4 mL) were added to EtOH (4 mL). Then, MPTS (200 μL) and NH_4_OH (40 μL) were added to the solution. The suspension was vigorously stirred at 700 rpm for 12 h at RT. After the reaction, the suspension was centrifuged and washed 3 times with EtOH. The final concentration of thiolated SiO_2_ (SiO_2_-SH) was adjusted to 50 mg·mL^−1^.

Ag NPs were attached on the SiO_2_-SH by reducing AgNO_3_ with octylamine in EG. PVP (5 mg) was dissolved in EG (25 mL). AgNO_3_ (26 mg) dissolved in EG (25 mL) was suspended in this PVP solution. SiO_2_-SH (30 mg) was then added to this suspension Octylamine (41.4 μL) was sequentially added and the suspension was vigorously stirred at 700 rpm for 1 h at RT. Then, the suspension was centrifuged and washed 5 times with EtOH.

#### 3.2.2. Synthesis of SiO_2_@Ag NPs with Silica Shells of Different Thicknesses

- Effect of the amount of the SiO_2_@Ag NPs on the silica shell thickness

Various amounts of the SiO_2_@Ag NPs (5, 10, and 20 mg) were dispersed in EtOH (1 mL). The SiO_2_@Ag NPs suspension was added in distilled water (15 mL) containing Na_2_SiO_3_ solution (14.4 μL). The suspension was stirred at 700 rpm for 1 h, and EtOH (60 mL) was added in the suspension, followed by stirring for 3 h. After stirring, the suspension was centrifuged at 8500 rpm for 15 min and washed by EtOH 3 times.

- Effect of the TEOS volume on silica shell thickness

SiO_2_@Ag NPs (10 mg) were dispersed in 1 mL of EtOH. The SiO_2_@Ag NPs suspension was added in distilled water (15 mL) containing Na_2_SiO_3_ solution (14.4 μL). The suspension was stirred at 700 rpm for 1 h, and EtOH (60 mL) was added in the suspension, followed by stirring for 3 h. Then, various volumes of TEOS (20, 40, and 60 μL) were added to the suspension under stirring. NH_4_OH (250 μL) was also added to the suspension, and the suspension was continuously stirred for 24 h. After stirring, the suspension was centrifuged at 8500 rpm for 15 min and washed 3 times with EtOH to remove excess reagents.

#### 3.2.3. Measurement of UV-Vis Absorption Spectra

The particles were dispersed in EtOH (2 mg·mL^−1^) and transferred to a cuvette. UV-Vis absorption of the sample was performed using a UV-Vis spectrophotometer (Mecasys OPTIZEN POP, Daejeon, Korea).

#### 3.2.4. Transmission Electron Microscopy (TEM) Imaging

The particles were dispersed in EtOH (2 mg·mL^−1^). Then, 10 μL of the sample was dropped on a copper grid (400 Mesh Cu, Pelco, Presno, CA, USA) and dried at RT. The size and morphology of the samples were observed by TEM (Libra 120, Carl Zeiss, Oberkochen, Germany).

#### 3.2.5. Measurement of the Size of the SiO_2_ NPs

The size of the nanoparticles and the thickness of the SiO_2_ shell were analyzed by digitalized measurement using Image J software (Bethesda, MD, USA). The average size of the NPs and the thickness of the SiO_2_ shell were calculated after analyzing at least 60 NPs.

## 4. Conclusions

In summary, the silica shell thickness of SiO_2_@Ag NPs was finely tuned in the range 5–40 nm by adjusting the number of SiO_2_@Ag NPs and the TEOS concentration. The silica shell thickness of SiO_2_@Ag NPs was found to be inversely proportional to the number of SiO_2_@Ag NPs and proportional to the volume of TEOS. Thin silica shells with thickness in the range 5.8–16.5 nm were formed on the surface of SiO_2_@Ag NP when the number of SiO_2_@Ag NPs decreased from 4.02 × 10^12^ NPs to 1.01 × 10^12^ NPs. In addition, the silica shell thickness increased from 23.5 to 40.1 nm when the final TEOS concentration was increased from 1.16 to 3.49 mM. In general, we obtained SiO_2_@Ag@SiO_2_ NPs with silica shell thicknesses of 5.8 ± 0.9, 9.7 ± 1.2, 16.5 ± 1.3, 23.5 ± 1.8, 32.6 ± 1.3, and 40.1 ± 2.1 nm by adjusting the number of SiO_2_@Ag NPs and the silica precursor volume. As expected, a thin silica shell coating on the surface of SiO_2_@Ag NPs does not seriously affect the plasmonic resonance properties of SiO_2_@Ag NPs. The successful coating of the thin and homogenous silica shell on the surface of SiO_2_@Ag NP was found to provide colloidal stability to the nanocomposite. The proposed technique is expected to be useful for understanding the distance-dependent electromagnetic field effects of SERS enhancement, MEF effect, and the quenching of complex NPs in sol-phase.

## Data Availability

Data is contained within the article and supplementary material.

## Supplementary Materials

The following are available online at https://www.mdpi.com/article/10.3390/ijms222111983/s1.
