# Supplementary material for: Ultra-Fine Control of Silica Shell Thickness on Silver Nanoparticle-Assembled Structures
Source: Int J Mol Sci. 2021 Nov 5;22(21):11983. doi: 10.3390/ijms222111983 (PMC8584519; doi:10.3390/ijms222111983)
Supplement: Supplementary file 1 [file ijms-22-11983-s001.zip › ijms-1432178-supplementary.pdf]

International Journal of *Molecular Sciences*

MDPI

Communication

Ultra-Fine Control of Silica Shell Thickness on Silver Nanoparticle-Assembled Structures

Eunil Hahm [1,†], Ahla Jo [1,†], Eun Ji Kang [1], Sungje Bock [1], Xuan-Hung Pham [1], Hyejin Chang [2,*] and Bong-Hyun Jun [1,*]

[1] Department of Bioscience and Biotechnology, Konkuk University, Seoul 05029, Korea; greenice@konkuk.ac.kr (E.H.), iamara0421@konkuk.ac.kr (A.J.), ejkang@konkuk.ac.kr (E.-J.K.), bsj4126@konkuk.ac.kr (S.B.), phamricky@konkuk.ac.kr (X.-H.P.)

[2] Division of Science Education, Kangwon National University, Chuncheon 24341, Korea

* Correspondence: hjchang@kangwon.ac.kr (H.C.), bjun@konkuk.ac.kr (B.-H.J.); Tel.: +82-2-450-0521 (B.-H.J.)

† E.H. and A.J. contributed equally to this work.

Supplementary materials

1. Characterization of the SiO_2@Ag NPs with the Silica Coating

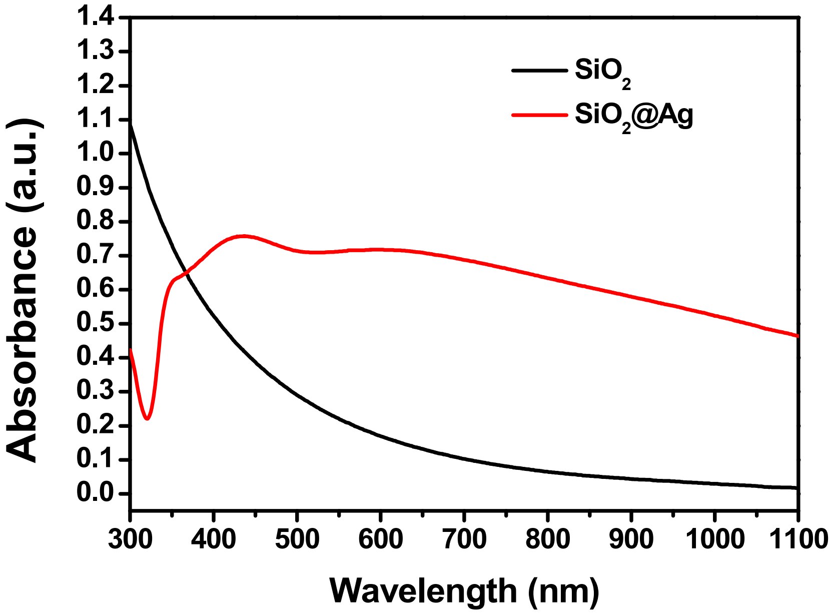

Figure S1. UV-vis spectra of SiO_2 and SiO_2@Ag NPs.

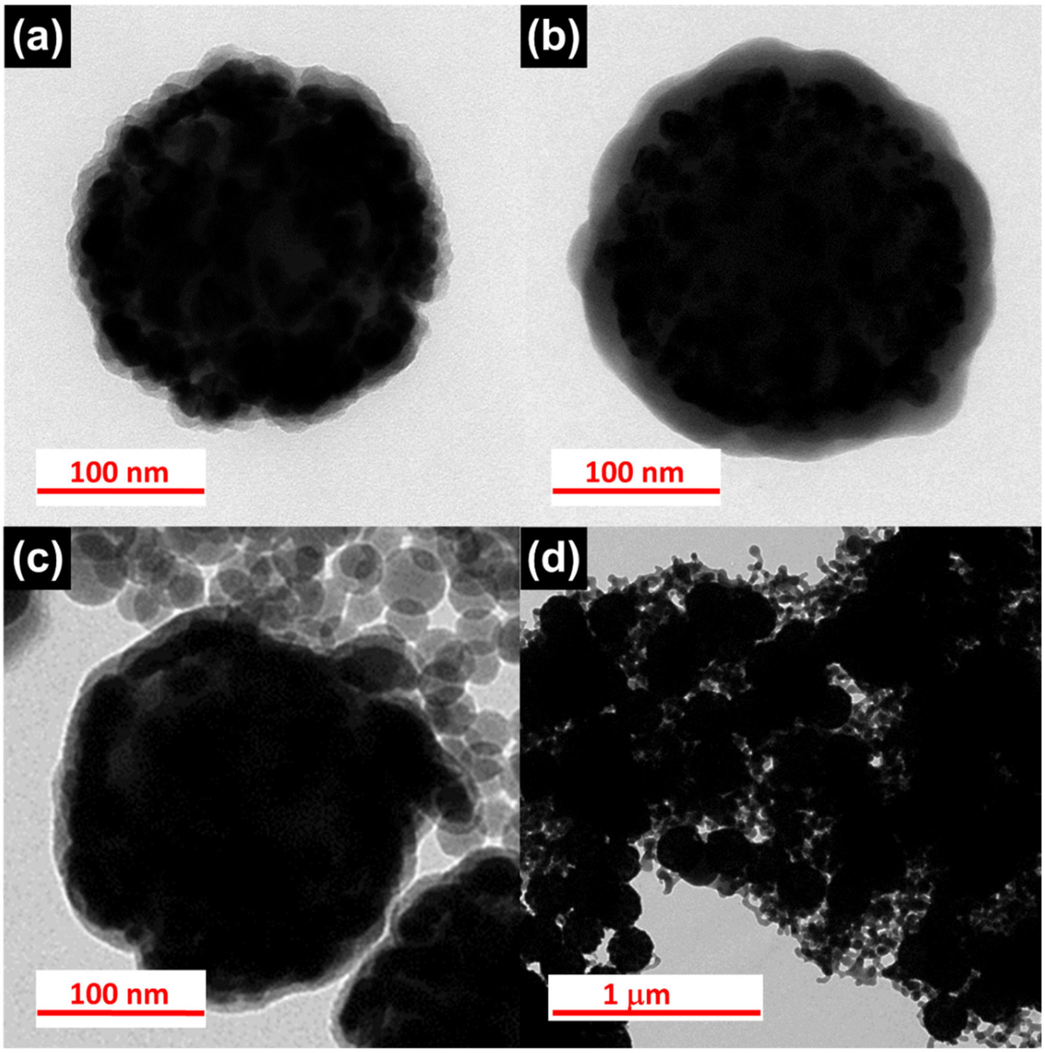

Figure S2. TEM images of SiO_2@Ag@SiO_2 NPs prepared by using Na_2SiO_3 with the following concentrations: (**a**) 45 μmol, (**b**) 90 μmol, (**c**) 180 μmol, and (**d**) 360 μmol.

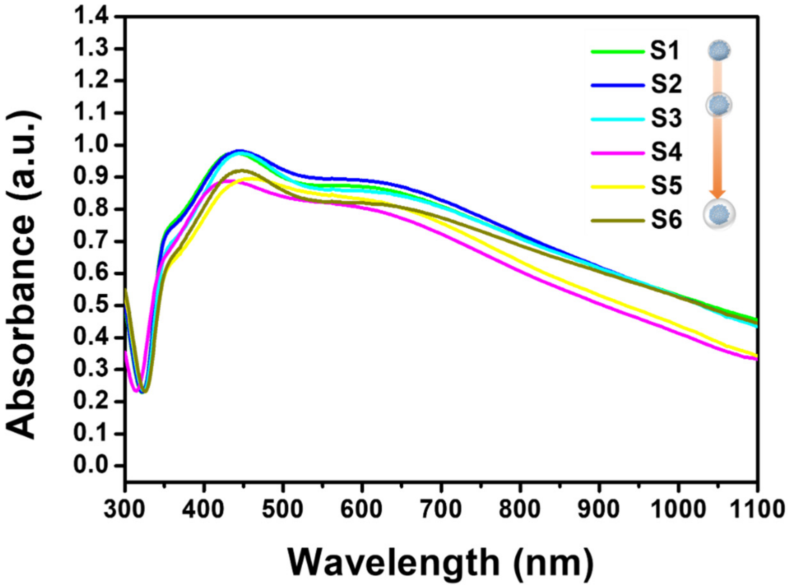

Figure S3. UV-Vis spectra of SiO_2@Ag@SiO_2 NPs with various silica shell thicknesses prepared under different conditions (S1–S6).

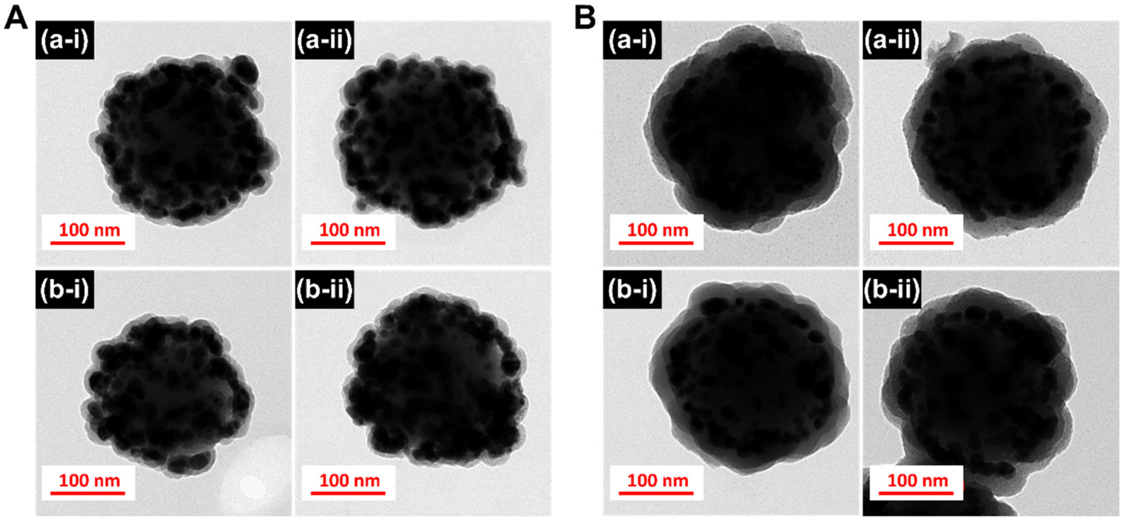

Figure S4. TEM images of SiO_2@Ag@SiO_2 NPs prepared by A) Na_2SiO_3 and B) Na_2SiO_3 + TEOS stored in (a) EtOH and (b) water for (i) 0 days and (ii) 10 days.

2. Calculation of The Number of Silica NPs and the Silica Shell Thickness on SiO_2@Ag NPs

2.1. Calculation of the Number of Silica NPs

The transmission electron microscopy (TEM) images reveal that the SiO_2 NPs usually have a spherical shape (Figure 1a). The silica nanoparticles were assumed to be perfectly spherical, and the volume of the silica nanoparticle was calculated from the measured diameter (Table S1). The mass of one SiO_2 NP is then calculated using the density of SiO_2 and the volume of one SiO_2 NP. Based on this result, the number of SiO_2 NPs per milligram is calculated as shown in Table S1.

Table S1. Experiment parameters of silica nanoparticle number, Na_2SiO_3 and TEOS concentration in S1–S6.

	Weight of SiO_2 (mg)	Number of NPs	Na_2SiO_3 (μL)	Na_2SiO_3 (μmol)	TEOS (μL)	TEOS (mM)
S1	20	4.024 x 10^{12}	14.4	90	-	-
S2	10	2.012 x 10^{12}	14.4	90	-	-
S3	5	1.006 x 10^{12}	14.4	90	-	-
S4	10	2.012 x 10^{12}	14.4	90	20	1.16
S5	10	2.012 x 10^{12}	14.4	90	40	2.33
S6	10	2.012 x 10^{12}	14.4	90	60	3.49

2.2. Calculation of the Number of Silica NPs and the Silica Shell Thickness on SiO_2@Ag NP

We assumed that the added Na_2SiO_3 reacted completely and converted to SiO_2.

As the Ag NPs are irregularly attached to SiO_2 NPs, it is difficult to obtain the correct diameter and the volume of the SiO_2@Ag NPs. To measure the diameter of the SiO_2@Ag NPs, we assumed that the Ag NPs formed a smooth shell on the surface of the SiO_2 NPs, forming perfectly spherical SiO_2@Ag NPs.

The silica shell thickness was calculated by Equation 1

$$\mathrm{SiO_2\ shell\ thickness} = \frac{\left[2 \times \sqrt[3]{\left\{\left(\frac{\mathrm{n} \times \mathrm{M}}{\rho \times \mathrm{N}} + V\right) \times \frac{3}{4\pi}\right\}} - \mathrm{d}\right]}{2} \quad (1)$$

where n is the number of moles of Na_2SiO_3 or TEOS, M is molecular weight of SiO_2, ϱ is the density of SiO_2, N is number of NPs, V is the volume of SiO_2@Ag, and d is the diameter of SiO_2@Ag.

The calculated silica shell thickness, the observed silica shell thickness, and their ratio are shown in Table S2.

Table S2. Diameter and shell thickness of SiO_2 NPs, SiO_2@Ag NPs and S1-S6 (SiO_2@Ag@SiO_2 NPs).

	**Diameter (nm)**	**Shell thickness (nm)**		**a/b**
		**Observed [a]**	**Calculated [b]**	
SiO_2	153±2.4			
SiO_2@Ag	192±7.5			
S1, SiO_2@Ag@SiO_2		5.8±0.9	4.2±0.2	1.38
S2, SiO_2@Ag@SiO_2		9.7±1.2	8.1±0.3	1.20
S3, SiO_2@Ag@SiO_2		16.5±1.3	15.0±0.5	1.10
S4, SiO_2@Ag@SiO_2		23.5±1.8	16.4±0.5	1.43
S5, SiO_2@Ag@SiO_2		32.6±1.3	22.4±0.6	1.46
S6, SiO_2@Ag@SiO_2		40.1±2.1	27.8±0.6	1.44

[a] Observed silica shell thickness. [b] Calculated silica shell thickness.
